# Pre-hospital exposures to antibiotics among children presenting with fever in northern Uganda: a facility-based cross-sectional study

**DOI:** 10.1186/s12887-022-03375-2

**Published:** 2022-06-01

**Authors:** Richard Nyeko, Felix Otim, Evelyn Miriam Obiya, Catherine Abala

**Affiliations:** 1 Department of Paediatrics and Child Health, Faculty of Medicine, Lira University, Lira, Uganda; 2 Department of Laboratory, St. Mary’s Hospital Lacor, Gulu, Uganda; 3 Department of Paediatrics and Child Health, St. Mary’s Hospital Lacor, Gulu, Uganda

**Keywords:** Antibiotic, Fever, Children, Antimicrobial resistance

## Abstract

**Background:**

The rise in the indiscriminate use of antibiotics has become a major global public health problem and presents the biggest global health challenge in the twenty-first century. In developing countries, caregivers initiate treatment with antibiotics at home before presentation to a health facility. However, there is a paucity of evolving data towards surveillance of this trend in low-income countries. We investigated antibiotic use among febrile children presenting to a tertiary health facility in northern Uganda.

**Methods:**

We conducted a cross-sectional study in a tertiary health facility in northern Uganda between March and September 2021. Children aged 6–59 months with fever were selected using systematic random sampling. A pre-tested interviewer-administered questionnaire was used the collect clinical data from the caregivers. Data were analyzed using SPSS version 23. Descriptive statistics and multiple logistic regression models were applied. *P*-value < 0.05 was considered for statistical significance.

**Results:**

Eighty-three (39.5%) of the 210 children with fever in this study used antibiotics prior to the hospital visit, 55.4% of which were on a self-medication basis, while 44.6% were empiric prescriptions. The most commonly used antibiotics were amoxicillin 33/83 (39.8%), erythromycin 18 (21.7%), metronidazole 14 (16.9%), ciprofloxacin 13 (15.7%) and ampicillin 6 (7.2%). The main sources of the antibiotics included buying from drug shops 30/83 (36.1%), issuance from clinics (33.7%), remnants at home (12.0%), picking from a neighbour (7.2%) and others (10.8%). The factors associated with antibiotic use among the febrile children were residence (*p* < 0.001); distance from the nearest health facility (*p* = 0.005); caregivers’ gender (*p* = 0.043); cough (*p* = 0.012); diarrhoea (*p* = 0.007); duration of fever (*p* = 0.002); perceived convulsion complicating fever (*p* = 0.026), and caregivers’ perception that fever (*p* = 0.001), cough (*p* = 0.003), diarrhoea (*p* < 0.001) and any infection (*p* < 0.001) are indications for antibiotics.

**Conclusions:**

Inappropriate use of antibiotics for childhood febrile illnesses is prevalent in the study setting, facilitated by the ease of access and use of leftover antibiotics. There is a need to address communities’ health-seeking behaviour and the health providers’ practice alike.

## Background

The rise in the use of antibacterial agents and the indiscriminate use of antibiotics has become a major global public health problem [1–3] and presents the biggest global health challenge in the 21^st^ century [4]. The World Health Organization (WHO) estimates that more than 50% of the global antibiotic use is inappropriate [5], being purchased privately without a prescription from pharmacies, drug shops or the informal sector – a practice shown to be associated with a parallel increase in development and spread of resistant bacterial strains [6].

Given the burden of infectious causes of illnesses and the health system challenges in most low-resource countries, the WHO Integrated Management of Childhood Illness (IMCI) treatment guidelines have been developed to guide the treatment of febrile childhood illnesses and the use of antibiotics in primary healthcare settings [7, 8]. The guidelines discourage the use of antibiotics in children with a common cold (viral upper respiratory tract infections) and acute watery diarrhoea.

Nonetheless, over half of all viral upper respiratory tract infections and viral diarrhoea cases in developing countries are reported to receive antibiotics inappropriately [9]. In these settings, the majority of caregivers initiate treatment of their children at home before presentation to a health facility [10–12] and is a common cause of self-medication with antibiotics. Caregivers assume the crucial role of undertaking the primary diagnosis, assessing the severity and making the decisions to seek or not to seek healthcare and initiate treatment, including use of antibiotics [13], notwithstanding that over 97% of febrile infants and young children have a self-limiting viral infection and therefore do not require antibiotics [14]. Help is only sought thereafter when the fever is unremitting or if complications set in [12, 15, 16].

This has been contributed to by many factors, including caregivers’ strong feelings of apprehension about the dangers of fever, enhanced by the relative ease with which communities access these medicines over-the-counter [3].

Uganda, just as many low-income countries, classifies antibiotics as prescription-only-medicine [17], but this is largely not enforced, permitting the public unlimited access to a variety of antibiotics that otherwise is provided on prescription [18, 19]. This potentially contributes to the growing practice of self-medication with antibiotics which exposes individuals and the population to several health risks including delays in seeking appropriate medical care when needed and the development of antimicrobial resistance [20]. Establishing the pattern of antibiotic use is thus crucial in understanding the epidemiology of resistance and launching initiatives to preserve the efficacy of antibiotics [21, 22]. We, therefore, determined the prevalence and predictors of pre-hospital exposures to antibiotics among febrile children presenting to a tertiary health facility in northern Uganda.

## Methods

### Study design and setting

We used a cross-sectional study design and systematic random sampling to select participants and collect quantitative data during a period of seven months from March 2021 to September 2021. Children aged 6-59 months with documented fever at presentation were included in the study, while those with known chronic illnesses taking recommended routine antibiotic prophylaxis, including children with sickle cell anaemia, cardiac diseases and any other child in such a category were excluded. The inclusion was stable during the study period. The study was conducted in the Paediatric outpatient department of St. Mary’s hospital Lacor in northern Uganda, Gulu district, located approximately 330km north of Kampala, Uganda’s capital city. This is a private not-for-profit faith-based 482-bed capacity facility with a high patient load and a wide catchment area, receiving on average, an annual outpatient attendance of children aged below six years of 22,142 [23]. Access to health services remains a challenge in the study setting and over 37% of the population moves a distance of more than 5 km to reach health services.

### Study population and sample size estimation

The study population comprised children aged 6-59 months with fever presenting to the tertiary health facility during the study period. A sample size of 210 participants was estimated using the single population proportion formula based on a proportion of antibiotic exposure (P) of 83.7% [14], with a marginal error (D) of 5%, a standard normal value (Z) corresponding to 95% certainty (1.96).

### Study procedures

A pre-tested semi-structured questionnaire was used to collect socio-demographic data, pre-hospital medications, source of medicines, reasons for pre-hospital medication, and the child’s related symptoms. The questionnaire was translated and administered in the most understandable way possible in the local language commonly spoken in the region/study setting. Pre-hospital exposures to antibiotics among children presenting with fever were classified into two basic categories as a) self-treatment if the child was treated without professional consultation or prescription, and b) empiric prescription if the caregiver sought care from a clinic and then received a prescription of antibiotics without established indications.

### Outcome variables

The outcome variable was the proportion of febrile children with pre-hospital exposure to antibiotics, while the predictor variables included the child’s and caregivers’ demographic characteristics, the child’s related symptoms, caregivers’ perceived cause and complications of fever, and perceived indications of antibiotics.

### Data management and statistical analysis

Data were analyzed using Statistical Package for Social Sciences (SPSS) software package (SPSS for Windows, Version 23.0. Chicago, SPSS Inc.). Descriptive statistics were used to summarize categorical variables as proportions and continuous variables as means and median. The Chi-square test or Fischer’s exact test (for categorical variables) and Student t-test (for continuous variables) with Odds ratios and 95% confidence intervals were performed to determine the association between the predictor variables and pre-hospital exposures to antibiotics.

The multivariate logistic regression model with the backward stepwise method was used to assess the factors that independently predicted pre-hospital antibiotic use and reported by an adjusted odds ratio (AOR) at a 95% confidence level. *P*-value <0.05 was considered for statistical significance. Variables that were statistically significant at the bivariate level (*p*<0.05) and those with *p* values <0.2 were included in the multivariate model.

## Results

### Description of the study population

**Fig. 1:**
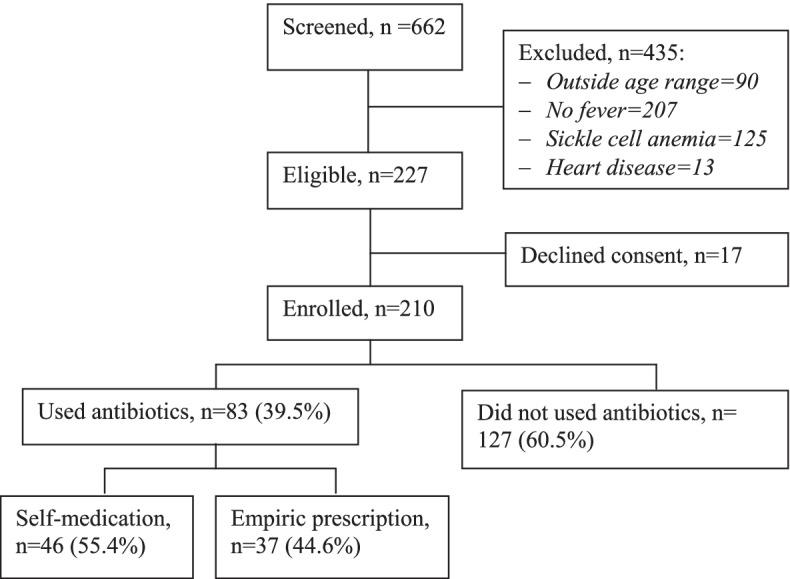
Study profile

During the study period (March 2021 to September 2021), a total of 210 children under the age of five years who presented to the paediatric outpatient clinic had documented fever and were recruited into the study (Fig. 1) - the majority of whom were treated as outpatients.

The majority of the children were males 56.2% (118/210) with a mean age of 27.3±16.9 months. One hundred and thirty-one (62.4%) of the participants were residing in rural settings and the majority (86.7%) lived within less than 5 km from the nearest health facility (Table 1).

**Table 1:** Demographic characteristics of the study population

**Variable**			**N**	**%**
**Child characteristics**				
Age (months)	Mean 27.3 (SD16.9)	Range (6–59)		
0–11			37	17.6
≥ 12			173	82.4
Gender				
Male			118	56.2
Female			92	43.8
Address				
Rural			131	62.4
Urban			79	37.6
Nearest health facility				
Hospital			62	29.5
Health centre			148	70.5
Distance nearest health facility				
˂5 km			182	86.7
≥ 5 km			28	13.3
**Caregiver characteristics**				
Gender				
Female			193	91.9
Male			17	8.1
Age (years)	Mean 30.0 (SD 7.9)	Range (18–60)		
≤ 30			135	64.3
˃30			75	35.7
Relation				
Mother			179	85.2
Father			16	7.6
Other			15	7.2
Education level				
None			20	9.5
Primary			89	42.4
Secondary			77	36.7
Tertiary			24	11.4
Occupation				
Employed			65	31.0
Unemployed			145	69.0

The majority of the caregivers were females 91.9% (193/210) and the biological mothers of the children 85.2% (179/210), while 7.6% (16/210) were the fathers, and 15 (7.2%) were other relations - comprising grandmothers 8 (3.8%), aunties 5 (2.4%) and siblings 2 (1.0%). The mean age of the caregivers was 30.0±7.9 years with a range of 18 to 60 years. One hundred and nine caregivers (51.9%) had primary or no education while 101 (48.1%) had secondary or tertiary education. The rest of the demographic characteristics were as shown in table 1.

### Clinical characteristics of the children presenting with fever

**Fig. 2:**
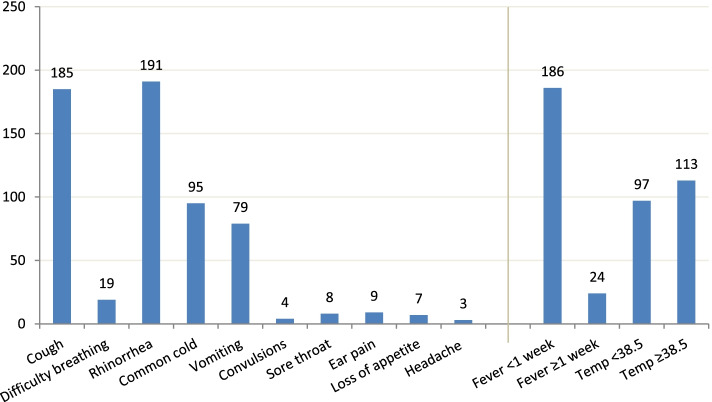
Associated symptoms among the children presenting with fever

The most common symptoms among children presenting with fever were common cold (rhinorrhea) 90.5% (191/210), cough 87.7% (185/210), diarrhoea 45.2% (95/210) and vomiting 37.6% (79/201) (Fig. 2). The majority of the children had a fever lasting less than 7 days 88.6% (186/201) and a presenting temperature of ≥38.5^0^C 53.8% (113/201). The rest of the associated symptoms are as shown in fig. 2.

### Caregivers’ perspective of fever

**Fig. 3:**
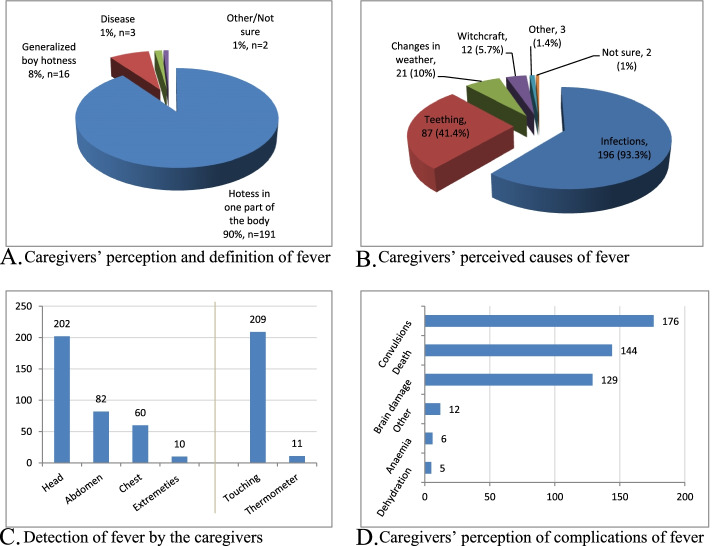
Caregivers’ perspectives of fever

The majority 191 (90.0%) of the caregivers perceived fever as hotness in only one part of the body and only 16 (8%) correctly defined fever as generalized hotness (Fig. 3A). An infection was perceived by the majority 196 (93.3%) of the caregivers as the cause of fever, in addition to a common belief among the respondents that fever is a result of teething, 87 (41.4%) (Fig. 3B). The most common method used to detect fever at home by the caregivers was by touch 209 (99.5%), with the head 202 (96.2%) being the most commonly felt area of the body (Fig. 3C). Convulsions 176 (83.8%), death 144 (68.6%) and brain damage 129 (61.4%) were the most feared consequences of fever in children among the respondents in the study setting (Fig. 3D). The rest of the caregivers’ perspectives regarding fever were as shown in Fig. 3.

### Prevalence of pre-hospital exposures to antibiotics among children with fever

**Fig. 4:**
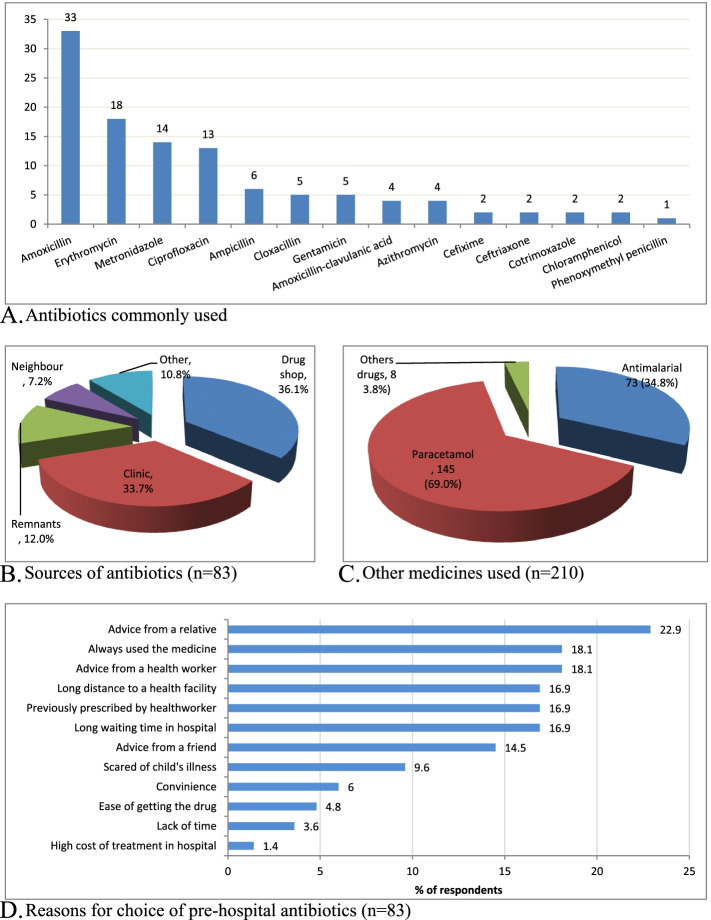
Antibiotics used, source of medications and reasons for choices among caregivers of children with fever

Of the 210 respondents, 157 (74.8%) had medications given to their children before presentation to the hospital, of which 83 (39.5%) used antibiotics. More than one half 46/83 (55.4%) of the antibiotics used were on a self-medication basis, while 44.6% (37/83) were empiric prescriptions from clinics and other health units. The most commonly used antibiotic was amoxicillin 33/83 (39.8%), followed by erythromycin 18 (21.7%), metronidazole 14 (16.9%), ciprofloxacin 13 (15.7%) and ampicillin 6 (7.2%) (Fig. 4A). The main sources of the antibiotics were; buying from the drug shops on a non-prescription basis 30/83 (36.1%), issuance from a clinic 28 (33.7%), remnants at home 10/83 (12.0%), antibiotics picked from a neighbour 6 (7.2%) and other sources 9 (10.8%) (Fig. 4B). The majority of the febrile children were also given paracetamol 145/210 (69.0%) and anti-malarial drugs 73 (34.8%) before presentation to the hospital (Fig. 4C). The main reasons for pre-hospital antibiotics use were, among others; advice from a relative 19/83 (22.9%), having always used the drug for febrile illnesses 15 (18.1%), advice from a health worker 15 (18.1%), long-distance to a health facility, the drug being previously prescribed by a health worker and long waiting time at health facility 14 (16.9%) each, and advice from a friend 12 (14.5%) (Fig. 4D). The rest of the medication use characteristics are as shown in fig. 4.

### Factors associated with pre-hospital exposures to antibiotics among children with fever

Children from rural settings were 3.7 times more likely to be given antibiotics for febrile illnesses prior to hospital visits than their urban counterparts, and this remained statistically significant at multivariate analysis (*p*<0.001, 95% CI 1.86-7.22) (Table 2). On the other hand, residing within a distance of less than 5 km from the nearest health facility was significantly independently associated with less likelihood of use of antibiotics during febrile illnesses prior to hospital visits (OR 0.26, 95% CI 0.10-0.68, *p*=0.005). Children whose nearest health facility was at the level of a hospital were less likely to be given antibiotics prior to a hospital visit, *p*<0.001 (OR 0.30, 95% CI 0.15-0.59), but this did not independently predict antibiotic use at multivariate analysis (*p*=0.226). Regarding caregivers’ characteristics, female caregivers and those aged ≤30 years were statistically significantly less likely to use antibiotics in children with fever prior to hospital visits than their counterparts who were male, *p*=0.007 (OR 0.24; 95% CI 0.08-0.72) or aged ˃30 years, *p*=0.014 (OR 0.49; 95% CI 0.27-0.87) respectively. However, only gender remained independently predictive of prior antibiotic use at multivariate analysis, *p*=0.043 (OR 0.30; 95% CI 0.09-0.96) (Table 2).

**Table 2:** Child and caregivers’ Demographic characteristics associated with pre-hospital antibiotic use

**Variable**	**Antibiotic use**		**Bivariate**		**Multivariate**^a^	
	**Yes *n* = 83 (%)**	**No *n* = 127 (%)**	**OR (95% CI)**	***P-value***	**aOR (95% CI)**	***P-value***
**Child characteristics**						
Age (months)						
0–11	16 (43.2)	21 (56.8)	1.21 (0.59–2.47)	0.612	-	
≥ 12	67 (38.7)	106 (61.3)				
Gender						
Male	50 (42.4)	68 (57.6)	1.32 (0.75–2.30)	0.338	-	
Female	33 (35.9)	59 (64.1)				
Address						
Rural	68 (51.9)	63 (48.1)	4.61 (2.38–8.90)	< 0.001	3.67 (1.86–7.22)	< 0.001
Urban	15 (19.0)	64 (81.0)				
Nearest HF						
Hospital	13 (21.0)	49 (79.0)	0.30 (0.15–0.59)	< 0.001	0.61 (0.27–1.37)	0.226
Health centre	70 (47.3)	78 (52.7)				
Distance						
˂5 km	62 (34.1)	120 (65.9)	0.17 (0.07–0.43)	< 0.001	0.26 (0.10–0.68)	0.005
≥ 5 km	21 (75.0)	7 (25.0)				
**Caregiver characteristics**						
Age (years)						
≤ 30	45 (33.3)	90 (66.7)	0.49 (0.27–0.87)	0.014	0.55 (0.29–1.04)	0.066
˃30	38 (50.7)	37 (49.3)				
Gender						
Female	71 (36.8)	122 (63.2)	0.24 (0.08–0.72)	0.007	0.30 (0.09–0.96)	0.043
Male	12 (70.6)	5 (29.4)				
Relation						
Parent	80 (41.0)	115 (59.0)	2.78 (0.76–10.18)	0.169	3.53 (0.91–13.64)	0.068
Guardian	3 (20.0)	12 (80.0)				
Education level						
≤ Primary	48 (44.0)	61 (58.0)	1.48 (0.85–2.59)	0.164	1.81 (1.00–3.29)	0.052
≥ Secondary	35 (34.7)	66 (65.3)				
Occupation						
Employed	23 (35.4)	42 (64.6)	0.78 (0.42–1.42)	0.410	-	
Not employed	60 (41.4)	85 (58.6)				

*aOR* adjusted Odds Ratio

^a^method = backward stepwise

Among the clinical symptoms, children who had a cough during their febrile illness were 5.6 times more likely to receive antibiotics prior to hospital visit compared to children who did not have a cough (*p*=0.001), and this remained statistically significant at multivariate analysis, *p*=0.012 (OR 5.12, 95% CI 1.43-18.32) (Table 3). Likewise, having diarrhoea, *p*=0.007 (OR 2.27, 95% CI 1.25-4.13) and fever lasting more than 7 days, *p*=0.002 (OR 4.87, 95% CI 1.79-13.24), were independently associated with increased likelihoods of using antibiotics prior to hospital visits.

**Table 3:** Symptoms characteristics and caregivers’ perceptions associated with antibiotic use

**Variable**	**Antibiotic use**		**Bivariate**		**Multivariate**^a^	
	**Yes *n* = 83 (%)**	**No *n* = 127 (%)**	**OR (95% CI)**	***P-value***	**aOR (95% CI)**	***P-value***
**Associates symptoms**						
Cough	80 (43.2)	105 (56.8)	5.59 (1.62–19.32)	0.001	5.12 (1.43–18.32)	0.012
DIB	10 (52.6)	9 (47.4)	1.80 (0.70–4.63)	0.226	-	
Common cold	78 (40.8)	113 (59.2)	1.93 (0.67–5.59)	0.206	-	
Diarrhoea	48 (50.5)	47 (49.5)	2.33 (1.33–4.11)	0.003	2.27 (1.25–4.13)	0.007
Vomiting	30 (38.0)	49 (62.0)	0.90 (0.51–1.60)	0.721	-	
Convulsions	2 (50.0)	2 (50.0)	1.54 (0.21–11.18)	0,649	-	
Throat/Ear pain	8 (47.1)	9 (52.9)	1.40 (0.52–3.78)	0.511	-	
Temp (≥ 38.5 °C)	40 (35.4)	73 (64.6)	0.69 (0.40–1.20)	0.187	0.60 (0.33–1.10)	0.100
Fever (≥ 1 week)	18 (75.0)	6 (25.0)	5.59 (2.11–14.76)	< 0.001	4.87 (1.79–13.24)	0.002
**Perceived cause of fever**						
Infections	78 (39.8)	118 (60.2)	1.19 (0.38–3.68)	0.761	-	
Teething	40 (46.0)	47 (54.0)	1.58 (0.90–2.78)	0.108	0.92 (0.43–1.97)	0.829
Weather changes	8 (38.1)	13 (61.9)	0.94 (0.37–2.37)	0.888	-	
Witchcraft	7 (58.3)	5 (41.7)	2.25 (0.69–7.34)	0.176	1.02 (0.19–5.49)	0.981
**Perceived fever complications**						
Convulsions	81 (46.0)	95 (54.0)	13.64 (3.17–58.68)	< 0.001	5.64 (1.23–25.91)	0.026
Brain damage	55 (42.6)	74 (57.4)	1.41 (0.79–2.50)	0.243	-	
Death	60 (41.7)	84 (58.3)	1.34 (0.73–2.45)	0.346	-	
Others	6 (40.0)	9 (60.0)	1.02 (0.35–2.99)	0.969	-	
**Perceived indications of antibiotics**						
Fever	55 (55.0)	45 (45.0)	3.58 (2.00–6.41)	< 0.001	3.58 (1.66–7.74)	0.001
Cough	67 (51.1)	64 (49.9)	4.12 (2.16–7.87)	< 0.001	3.54 (1.55–8.06)	0.003
Common cold	24 (48.0)	26 (52.0)	1.58 (0.83–3.00)	0.163	1.51 (0.60–3.79)	0.379
Diarrhoea	41 (77.4)	12 (22.6)	9.36 (4.49–19.49)	< 0.001	8.00 (3.31–19.30)	< 0.001
Bacterial infection	38 (46.3)	44 (53.7)	1.59 (0.91–2.81)	0.107	1.66 (0.75–3.71)	0.215
Any infection	33 (63.5)	19 (36.5)	3.75 (1.95–7.23)	< 0.001	8.21 (3.23–20.82)	< 0.001

*aOR* adjusted Odds Ration, *DIB* Difficulty in breathing, *Temp* Temperature

^a^method = backward stepwise

Caregivers’ fear of convulsions complicating fever in children was significantly associated with the use of antibiotics prior to a hospital visit at bivariate analysis (*p*<0.001), and this remained statistically significant at multivariate analysis, *p*=0.026 (OR 5.64; 95% CI 1.23-25.91). Likewise, caregivers who believed that fever, cough, diarrhoea or any form of infection are indications for antibiotics were significantly more likely to use antibiotics at bivariate analysis, *p*<0.001 respectively. These factors remained independently predictive of antibiotic use at multivariate analysis, where the perception of fever or cough as indications for antibiotics increased the likelihood of its use 3.5-fold, *p*=0.001 and *p*=0.003 respectively. Likewise, the perception that diarrhoea or any form of infection were indications for antibiotics was associated with an 8-fold likelihood for its use, *p*<0.001 respectively (Table 3).

## Discussion

The use of antibiotics prior to a hospital visit is a common practice in many resource-limited settings and a potential threat to antibiotics stewardship and resistance globally. The current facility-based study investigated pre-hospital antibiotic use among children presenting with fever at a tertiary health facility in northern Uganda and offers new insights and understanding of inappropriate antibiotic use among children in a resource-limited context.

### Prevalence of antibiotics use in children with fever prior to a hospital visit

The prevalence of pre-hospital exposure to antibiotics in this study was 39.5%, over half (54.5%) of which were on a self-treatment basis and 44.5% as empiric prescriptions from clinics or other health units. This finding is similar to the 40.1% antibiotic use by caregivers of children with fever reported in Kenya [24]. Our prevalence, however, is lower than the 70.5% and 69% previously reported in Uganda [25] and Vietnam [26] respectively, but higher than the 13.5% reported in an Iranian study [15]. The 39.5% rate of antibiotic use in the current study could, however, be an underestimate as evidenced by a recent finding indicating a low validity of caregivers’ reports on prior intake of antibacterials by their children (14.4% reported vs 63.7% antibacterial detection in blood and urine samples) [27]. It is also possible that the covid-19 pandemic could have had an impact on the level of antibiotic use, drawing from a recent report from Kampala, Uganda’s capital city, showing a general reduction in access to maternal and child health services during the covid-19-related lockdowns and restrictions [28]. However, it’s difficult to state with certainty in which direction this could have affected antibiotics use.

The high prevalence of antibiotic use in our study may not be surprising as most of the antibiotics in Uganda, just like in many low-income countries, can be bought from private pharmacies without prescription by qualified health providers. This is concerning and could present enormous challenges, including delayed healthcare seeking, exacerbating or masking symptoms, affecting the laboratory diagnostic results, as well as the development of antimicrobial resistance [29]. While antibacterial resistance will often develop, the risk is likely to be higher with inappropriate use and self-medication [30].

The antibiotics used prior to a hospital visit in the current study were mainly bought from the drug shops on a non-prescription basis, given from a clinic on empiric prescription, remnants at home, or picked from a neighbour. This finding resonates with that reported in China where one-third of caregivers used leftover antibiotics to treat fever and respiratory problems [31] and attests to an earlier finding in Uganda that pharmacies, drug shops, and clinics dispense whatever medicines the client requests [19]. The use of home remnants of antibiotics as found in this study should be concerning given the usually un-ideal storage conditions and risks of expiry, rendering the medicines ineffective due to loss of potency, besides toxic metabolites [32]. Further analysis in this study indicates that caregivers from rural settings were more likely to use leftovers of drugs in the house or pick drugs from their neighbours and friends, than the urban dwellers. This finding mirrors one previously reported in Nigeria - postulated to be a result of the homogenous nature of the rural residents with regards to culture as opposed to the urban dwellers who tend to be heterogeneous with differing cultural backgrounds [33]. The rural-urban difference may also be attributed to the fact that urban residents are more exposed to health educative information from the media, as well as the ease of access to primary healthcare facilities. In fact, in this study, the caregivers from the urban areas were more likely to be living within less than 5 km from the nearest health facility compared to their rural counterparts. The fact that the respondents in this study did not significantly, in their first actions, use the primary health centres meant to be the first points of contact might be that they were not easily accessible geographically or a reflection of the level of confidence they have in these lower public health facilities to manage their children’s conditions.

The main reasons advanced by the respondents for, largely inappropriate, pre-hospital use of antibiotics, included: advice from a relative, having always used the drug for febrile illnesses, advice from a health worker, long-distance to a health facility, the drug being previously prescribed by a health worker, and long waiting time in hospitals, among others. This finding seems to agree with that in earlier reports among adults presenting to hospitals in the study setting [3] and Malawi [29], where previous experience with the antibiotics informed patient’s choice for self-medication, despite their limited knowledge regarding the therapeutic indications and correct dosage schedule [29]. The influence of distance to a health facility and lengthy-time at health facilities on inappropriate use of antibiotics as found in this study resonates with findings reported in Tanzania [34] and Nigeria [10], and speak to the health system challenges in low-income countries. Importantly, our finding also implies that the inappropriate use of antibiotics can be blamed not on patients alone because the healthcare workers also played a contributory role through inappropriate advice and prescriptions. While the study covered only a short time period, we believe the results would not have vastly varied by time points since antibiotics use in Uganda is not believed to vary a lot with seasonality, being driven by the year-round high burden of infectious causes of morbidity coupled with the weak antibiotic stewardship.

### Antibiotics commonly used among children with fever

The antibiotics most commonly used by children under five years with fever, largely inappropriately, included amoxicillin, followed by erythromycin, metronidazole, ciprofloxacin and ampicillin. This finding mirrors that reported in a study in Lagos State University Teaching Hospital, Nigeria, where amoxicillin, ampicillin/cloxacillin, cotrimoxazole and Amoxil-clavulanic acid were the common antibiotics used for the treatment of children with fever [15]. Similarly, in Vietnam, ampicillin, amoxicillin, cephalosporins, sulphonamides, trimethoprim and macrolides were the most commonly used drugs for treating children with fever [13]. This finding has important implications on antibacterials in low-income countries and the heightened risk of antibacterial resistance. This study, however, was unable to ascertain the dosing of the antibiotics given to the children before the hospital visit. The ascertainment of dosing would be important given that in addition to predisposing to the development of resistance, inappropriate dosing of antibiotics could also lead to adverse outcomes - as pediatric doses are weight-based and differ significantly between age groups.

### Predictors of pre-hospital use of antibiotics among children with fever

This study brings to light significant differences in pre-hospital exposure to antibiotics with regard to several respondents’ characteristics. We observed a strong association between the respondents’ place of residence and pre-hospital exposure to antibiotics. Individuals residing in rural settings had a significantly higher likelihood of using antibiotics to treat children prior to a hospital visit than those from urban settings. This finding contrasts with that from an earlier report by Kibuule et al in Kampala, Uganda’s capital city, where households living in urban settings more likely used antibiotics than their rural counterparts [25]. We believe this difference could partly be contextual given the big contrast between the two settings.

Residing within less than 5km distance from the nearest health facility was significantly associated with less use of antibiotics prior to a hospital visit. This finding may not be unexpected since the distance from a health facility is an important determinant of access to healthcare – a reason advanced by the respondents for choosing to give treatment from home and speaks to the importance of improving access to healthcare services in low-income countries. Our study did not find a statistically significant association between the child’s age and antibiotics use, contrary to a report by Nguyen et al in Vietnam [26]. This might be that caregivers do not consider that the child’s age could have a bearing on the adverse effects of antibiotics.

Female caregivers of children with fever were significantly less likely to use antibiotics compared to their male counterparts, a finding which accords with that reported in the Kingdom of Saudi Arabia where men were more likely to self-prescribe antibiotics than females [35]. This could be attributed to the fact that females generally have better healthcare-seeking behaviour [36], a phenomenon supported by the high proportion of female caregivers found in this study. We did not find an independent association between caregivers’ age and use of antibiotics among children with fever, though caregivers aged 30 years and below had a 51% reduced chance of exposing their children to antibiotics prior to a hospital visit at bivariate analysis compared to those aged above 30 years. This finding reflects that reported in the Kingdom Republic of Saudi Arabia where the probability of self-prescription was higher among respondents older than 30 years compared to those aged 18-30 years [35], probably attributed to previous experiences that develop over time.

Symptom-wise, pre-hospital use of antibiotics was significantly dependent on the child’s symptoms and caregivers’ perceived indications for use of antibiotics. Children presenting to the hospital with a long duration of illness - denoted by fever lasting 7 days or more, were significantly more likely to be given antibiotics prior to a hospital visit. We believe, as postulated by Nguyen et al, this might be due to the perception that febrile illness of longer duration is more severe and requires antibiotics [26]. On the other hand, pre-hospital medication may, in itself be a cause for delay in health-seeking, consequently leading to more severe illness, delayed diagnosis, serious complications, and even death.

Our study also showed that children who had cough and diarrhoea during their febrile illnesses were significantly more likely to be given antibiotics compared to those who did not have these symptoms. This finding could relate to the respondents’ perception regarding the indications for antibiotics, given that in this study, caregivers who believed that fever, cough, diarrhoea and any form of infection are indications for antibiotics were significantly more likely to use the same. This relationship could explain the 39.5% observed rate of antibiotic use found in this study given that the majority of the children presenting with fever also had a cough (87.7%), common cold (90.5%) and diarrhoea (45.2%). In fact, 96.4% of the children who received antibiotics during their febrile illnesses in this study also had a cough. This finding correlates with reports from Nigeria [14], Uganda [25] and Vietnam [26] where upper respiratory tract infections (common cold) were a cause of up to 83.7, 70.5 and 63% antibiotics exposure among children respectively, and corroborates that reported among adult patients in the Kingdom of Saudi Arabia [35]. Similarly, in surveys conducted in rural China [31] and Indonesia [37], antibiotics were 42% more likely to be used if the child had a cough and/or common cold. Symptoms-driven use of antibiotics in the current study could reflect limited knowledge among the population regarding the therapeutic indications for antibiotics, just as reported in Malawi [29].

## Conclusions and recommendations

Inappropriate use of antibiotics for childhood febrile illnesses is prevalent in the study setting, facilitated by the ease of access to medicines through private drug outlets such as drug shops and clinics, as well as the use of leftover antibiotics. Caregivers’ perception regarding indications for antibiotics and health providers’ practice remain important contributors to increased use of antibiotics in children. Thus, there is a need to address both the communities' health-seeking behaviour and the health providers' practice alike to minimize the problem of inappropriate antibiotic use. This should encompass behaviour change educational interventions to the populace about the causes of fever, cough, common colds, and diarrhoea and their natural course and appropriate antibiotic indications.

### Limitations

Our findings could have been limited by recall and information bias given the reported low validity of reported antibiotic use. However, this was minimized by the use of a simple and easy-to-understand questionnaire, administered confidentially. The single-centre nature of the study could have also affected the external validity of the study findings since a multicentre study could not be carried out because of limited resources. The choice of the study site was, however, meant to circumvent this limitation since this is one of the largest tertiary health facilities in the region with a large catchment area, attracting patients from all over the region.

## Data Availability

The datasets used and/or analysed during the current study are available from the corresponding author on reasonable request.
